# Marine Vertebrates Impact the Bacterial Community Composition and Food Webs of Antarctic Microbial Mats

**DOI:** 10.3389/fmicb.2022.841175

**Published:** 2022-04-08

**Authors:** Pablo Almela, David Velázquez, Eugenio Rico, Ana Justel, Antonio Quesada

**Affiliations:** ^1^Department of Biology, Universidad Autónoma de Madrid, Madrid, Spain; ^2^Department of Ecology, Universidad Autónoma de Madrid, Madrid, Spain; ^3^Centro de Investigación en Biodiversidad y Cambio Global (CIBC-UAM), Universidad Autónoma de Madrid, Madrid, Spain; ^4^UC3M-Santander Big Data Institute (IBiDat), Universidad Carlos III de Madrid, Madrid, Spain; ^5^Department of Mathematics, Universidad Autónoma de Madrid, Madrid, Spain

**Keywords:** penguins, nitrogen, phosphorus, microbial mat, trophic relationships, bacterial community, Antarctica

## Abstract

The biological activity of marine vertebrates represents an input of nutrients for Antarctic terrestrial biota, with relevant consequences for the entire ecosystem. Even though microbial mats assemble most of the biological diversity of the non-marine Antarctica, the effects of the local macrofauna on these microecosystems remain understudied. Using 16S rRNA gene sequencing, ^13^C and ^15^N stable isotopes, and by characterizing the P and N-derived nutrient levels, we evaluated the effects of penguins and other marine vertebrates on four microbial mats located along the Antarctic Peninsula. Our results show that P concentrations, C/N and N/P ratios, and δ^15^N values of “penguin-impacted” microbial mats were significantly higher than values obtained for “macrofauna-free” sample. Nutrients derived from penguin colonies and other marine vertebrates altered the trophic interactions of communities within microbial mats, as well as the relative abundance and trophic position of meiofaunal groups. Twenty-nine bacterial families from eight different phyla significantly changed with the presence of penguins, with inorganic nitrogen (NH_4_^+^ and NO_3_^–^) and δ^15^N appearing as key factors in driving bacterial community composition. An apparent change in richness, diversity, and dominance of prokaryotes was also related to penguin-derived nutrients, affecting N utilization strategies of microbial mats and relating oligotrophic systems to communities with a higher metabolic versatility. The interdisciplinary approach of this study makes these results advance our understanding of interactions and composition of communities inhabiting microbial mats from Antarctica, revealing how they are deeply associated with marine animals.

## Introduction

The Antarctic climatic conditions impose severe restrictions on living organisms. Low temperatures, low availability of liquid water, long periods with continuous irradiance, and months of nearly complete darkness during winter (Thomas et al., 2008) result in an ecosystem dominated by microorganisms. The harsh environmental conditions, together with the prevalence of rocky sites with low nutrient levels and continental geographic isolation, make microscopic organisms the most diverse and abundant components of terrestrial Antarctic communities (Hughes et al., 2015). This provides a unique setup to validate ecological hypotheses on microorganism-based food webs in a more “controlled” scenario (Hansson and Tranvik, 2003). Cyanobacteria become key players at these high-latitude ecosystems (Quesada and Vincent, 2012), because of their extreme resilience resisting desiccation, freeze–thaw cycles, and high levels of UV radiation, which contribute to their dominance in polar aquatic ecosystems (Bonilla et al., 2005). Cyanobacterial microbial mats accommodate high biodiversity and represent one of the highest concentrations of non-marine biomass with a ubiquitous distribution throughout Antarctica (Quesada and Vincent, 2012) and therefore constitute an important carbon (C) reservoir.

Recently, Velázquez et al. (2017) and Almela et al. (2019) described in detail the trophic relationships among the different elements of the community within Antarctic microbial mats. The authors described at least four trophic levels in the microbial mats and a community adapted to a short growing season by virtue of a fine temporal coupling, where fungal and bacterial activities represent the main connectors between consumers and producers. These studies were conducted from communities growing in oligotrophic environments. However, microbial mats are also found in the vicinity of areas with presence of marine vertebrates. The influence of local macrofauna on polar ecosystems is well known (Jakubas et al., 2008; Zawierucha et al., 2016, 2019; Bokhorst et al., 2019; Wang et al., 2020), but the influence of their biological activity on the ecology of microbial mats (diversity and trophic interactions) is understudied. Seal haul-outs and seabird colonies on Antarctic coastal ecosystems are not considered part of the terrestrial biota (Laybourn-Parry, 2009), but they influence the non-marine ecosystem where they remain a considerable portion of their time. The overall seabird population in Antarctica and its sub-Antarctic islands represents 26% of the global population. However, the nesting populations in this regions account for 80% of the total N and P excreted worldwide (Otero et al., 2018). Therefore, marine vertebrates, mainly seabirds, and especially penguins, provide a relevant input of nutrients through their droppings into this ecosystem by transferring nitrogen and phosphorus from sea to land (Bokhorst et al., 2019; Wang et al., 2020), which could deeply modify the ecosystem where they reside (De La Peña-Lastra, 2021).

The Antarctic Peninsula region has shown rapid warming, similar in magnitude to some regions in the Arctic and substantially faster than the global mean (Robinson et al., 2020). This region is considered as one of the most sensitive on Earth to climate warming as average temperatures are close to the freezing point (Rochera et al., 2013), and small temperature shifts can trigger pronounced effects. Climate change in the region is already causing an alteration in marine vertebrates’ communities with shifts in species and abundance in their populations (Lynch et al., 2012; Clucas et al., 2014; Dunn et al., 2016; Morley et al., 2019; Ropert-Coudert et al., 2019), suggesting major implications for local terrestrial biodiversity patterns in Antarctica (Bokhorst et al., 2019).

Here we determined δ^15^N, phosphorus (P), total nitrogen (TN), C/N, N/P, and inorganic nitrogen (N-NH_4_^+^ and NO_3_) in microbial mats adjacent and isolated from penguin colonies along the Antarctic Peninsula. The aim of this study was to identify the impact of N and P derived from macrofaunal activity on benthic microbial mats and hence its effects on bacterial, fungal, photosynthetic, and meiofaunal communities and the trophic relationships that occur within them. To date, it is unknown how the activity of marine vertebrates affects these polar microecosystems. The results presented here will shed new insights on microbial community function to understand and assess the ecological risks over freshwater microbial communities derived from changes on populations of seabirds and marine mammals.

## Materials and Methods

### Study Site and Sampling

**FIGURE 1:**
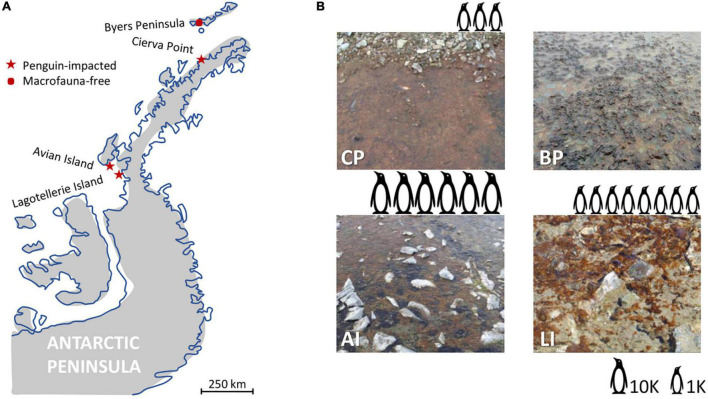
Map indicating the location of the sampling areas along the Antarctic Peninsula. **(A)** Distribution of the sampled microbial mats along the Antarctic Peninsula. Red stars indicate the sampling areas with penguin colonies in their surroundings, whereas the red dot indicates the sampling area isolated from marine vertebrates. **(B)** Detailed picture of microbial mats from Byers Peninsula Plateau (BP), Caleta Primavera (CP), Avian Island (AI), and Lagotellerie Island (LI). Images of penguins represent the population (number of nests) of penguin colonies from CP, AI, and LI sampling areas (data from MAPPPD; Humphries et al., 2017).

Samplings were conducted during two different Antarctic campaigns in different ice-free areas of the Antarctic Peninsula (Figure 1A): Byers Peninsula plateau (BP), Cierva Point (CP), and Avian Island (AI) microbial mats were sampled in February 2016 and Lagotellerie Island (LI) microbial mat in February 2019.

BP is one of the largest ice-free areas in the Antarctic Peninsula. It is located at the western end of Livingston Island (South Shetland archipelago). It is designated as the Antarctic Specially Protected Area (ASPA) no. 126 to protect its terrestrial and lacustrine habitats, and it is considered one of the main Antarctic hotspots of biodiversity (ATCM, 2011). Here, the sampled microbial mat (BP) was located over the plateau, 1.5 km away from the coast and 60 masl. The microbial mat was multilayered and orange pigmented, with an average thickness of 13 ± 2 mm. Mosses were observed at the edge of the waterbody where the microbial mat was collected, but sporadically, some stems can be observed within the mat structure.

CP, which is also designated as ASPA (no. 134), is located in the northwestern portion of the Antarctic Peninsula. The site comprises the ice-free area between the southwest coast of Cierva Cove and the northeast coast of Santucci Cove. It has great scientific value because of its unusual biodiversity, which includes numerous species of birds, flora, and invertebrates (ATCM, 2013). The sampled microbial mat (CP), orange–green in color and 4 ± 1 mm in thickness, was located within the ASPA, 200 m away from the coast and 34 masl.

AI (ASPA no. 117) is situated 400 m south of Adelaide Island at Marguerite Bay on the western side of the central Antarctic Peninsula. The island comprises 0.49 km^2^, which usually remains ice-free during the summer seasons. The values described under its ASPA designation aim to protect the abundance and diversity of breeding seabirds on the island (ATCM, 2002). The sampled microbial mat (AI), purple–green in color and 5 ± 2 mm in thickness, was collected in a pond at less than 30 m from the coast and 10 masl.

Finally, we also collected samples from LI, located at Marguerite Bay as well. It has been designated as ASPA no. 115 because of its relatively diverse flora and fauna, which is characteristic of the southern Antarctic Peninsula (ATCM, 2017). The studied microbial mat from this location, orange–brown in color and 5 ± 2 mm in thickness, was collected in an outlet stream to the coast at 2 masl.

The presence of penguins in the surroundings of the studied microbial mats was different among the sampling areas. On the one hand, CP mat was adjacent to an active Gentoo penguin (*Pygoscelis papua*) colony (ATCM, 2013), and AI and LI mats were adjacent to an Adelie penguin (*Pygoscelis adeliae*) colony (ATCM, 2002, 2017). Penguin population data were obtained from the online database Mapping Application for Penguin Populations and Projected Dynamics (MAPPPD; Humphries et al., 2017), estimating colonies of 3304 (CP), 59,895 (AI), and 8195 (LI) nests (Figure 1B). These locations have a remarkable influence of different seabirds, such as skuas (*Catharacta maccormicki, Catharacta loennbergi*), gulls (*Larus dominicanus*), and imperial shags (*Phalacrocorax atriceps*). Also, southern elephant seals (*Mirounga leonina*) and seals (*Arctocephalus gazella, Leptonychotes weddellii*) are frequently observed in the surroundings (ATCM, 2002, 2013, 2017). On the other hand, the BP sample was collected from an area with no influence of marine vertebrates, far and geographically separate from the coastline, and therefore without nutrient inputs derived from macrofaunal activity. The four sampling areas are not subjected to almost any direct human influence, and visitors can only access the sites for scientific purposes and with permits. Both access and sampling were specifically permitted by Spanish Polar Committee.

All samples were collected as follows: three replicates of approximately 6 cm^2^ were haphazardly collected using sterilized metal spatulas from each sampling spot, covering a sampling area of approximately 2 m^2^ of the wetland. These samples were stored in sterile Whirl-Pak bags (Nasco Ltd.) at −20°C until further analysis at the laboratory.

### Environmental Data Collection and Chemical and Stable Isotope Analyses

The averages of 21 years of number of days per year with temperatures above freezing point (TAF) were considered a good proxy to summarize the climatic characteristics of the sampling sites. This parameter was estimated with the hourly data obtained from the Modern-Era Retrospective Analysis for Research and Applications (Rienecker et al., 2011).

P is an important element of penguin feces, which together with N, and its high δ^15^N signature, are usually much higher in areas influenced by penguins than the values registered in non-affected sites (Liu et al., 2013; Otero et al., 2018; Bokhorst et al., 2019; Wang et al., 2020). Analyses of P, TN, and δ^15^N values and C/N and N/P ratios were carried out to assess the microbial mat nutrient levels and whether they are significantly affected by the biological activity of penguins and other marine vertebrates. Briefly, fresh material from each microbial mat was dried out for 48 h at 65°C and homogenized after sieving through a 0.5-mm strainer. Therefore, the nutrient content was jointly determined for the biomass and the contents in the interstitial water present in the samples and expressed as mg ⋅ g^–1^. Triplicates of 0.3 g of each dried sample were digested with HNO_3_ and HCl (10 mL) at 105°C for 2 h. After filtration through a 0.45-μm nylon filter membranes, the P concentration was determined by inductively coupled plasma emission spectroscopy (iCAP 6500 DUO; Thermo Scientific, Waltham, MA, United States). The bulk C and N concentrations of samples were measured in triplicate by a LECO CHNS-932 Analyzer (model no. 601-800-500). Briefly, approximately 10 mg of each dried sample was placed in a tin cup, wrapped to compact it, and then analyzed. C/N and N/P ratios were calculated from the C, N, and P concentrations.

The δ^15^N signature of each microbial mat was quantified by triplicate using an isotope ratio mass spectrometer (MEIR; 20-20 PDZ Europa mass spectrometer, Sandbach, United Kingdom), from fresh samples placed in 175-μL zinc cases. Isotopic values were expressed as δ^15^N = [(*R* sample/*R* standard) − 1] × 1000 (‰), where *R* represents the ^15^N/^14^N ratio, and the *R* standard value was based on atmospheric N (N_2_-atm).

NO_3_^–^ and N-NH_4_^+^ concentrations were analyzed to further study the effect of penguin-derived N on microbial mats. NO_3_^–^ was determined by liquid phase chromatography. Briefly, 2 g of dried and sieved material from each sample was extracted by an aqueous solution (50 mL final volume) while stirring for 30 min. Extracts were filtered through 0.45-μm nylon membranes and then retained in the cartridges (Metrosep A Supp 5-250; Metrohm, Herisau, Switzerland), eluted, and analyzed by ion chromatography (883 Basic IC; Metrohm, United States). The nitrogen content in ammonium (N-NH_4_^+^) was determined by ion chromatography following the same previously described protocol (in this case, boiling while stirring for the extraction), but the extracts were retained in cartridges Metrosep C6 250/4.0 (Metrohm, Herisau, Switzerland). Estimations of total biomass were calculated from the ash free dry weight (AFDW) gravimetric approach. Briefly, triplicates of 8-mm-diameter cores per sample of fresh material were dried, weighed, ashed in a muffle furnace at high temperature (450°C for 4 h), and reweighed. The loss is referred to as AFDW and reflects the C content. The biomass values were expressed as total organic C (TOC) per surface (mg ⋅ cm^–2^).

### Phototrophic and Fungal Community Estimations

Phototrophic community abundance (Cyanobacteria, Chlorophyta, Bacillariophyta, and other algae) was estimated by chlorophyll-*a* (chla) concentration. Chla was extracted in triplicates from 8 mm in diameter core samples, using 90% (vol/vol) acetone (Ritchie, 2008). Extracts were measured spectrophotometrically (Hitachi U-2000 Spectrophotometer; Hitachi Ltd., Tokyo, Japan) at 665 nm.

Ergosterol, a biochemical marker of fungal active biomass, was quantified using high-performance liquid chromatography equipped with a UV detector (282 nm) as described by Gessner (2020). Extraction was performed by triplicate according to Romaní et al. (2009) using samples from the 8-mm-diameter core. The concentration of ergosterol in the eluted product was then transformed to fungal biomass, assuming that 5.5 mg of ergosterol was found in 1 g of fungal biomass (Gessner and Chauvet, 1993).

### Microscopic Identification of the Community Composition and ^13^C and ^15^N Natural Abundance Analysis

The composition of the trophic community was studied from 8-mm-diameter core samples of fresh material, which were manually disaggregated. Relative abundance of the meiofauna (rotifers, tardigrades, and nematodes) was analyzed by using a stereo-zoom microscope (Leica MZ75) and interpreted per surface units. Photosynthetic organisms were determined using epifluorescence microscopy equipped with an Olympus^®^ blue filter set (EF 400–490 nm, DM 570, FB 590) and an Olympus^®^ green filter set (EF 530–545 nm, DM 570, FB 590) to excite chla and cyanobacterial phycobilin, respectively.

Stable isotope ^13^C and ^15^N provide information on the original nutrient source, which makes it one of the most helpful tools for assessing food web functioning. For δ^13^C‰ and δ^15^N‰ natural abundance signal analysis, individuals of different trophic levels were manually separated under the microscope by microdissection and encapsulated in triplicates in 175-μL zinc cases and dried at 65°C for 48 h. A minimum of 0.02 mg of dry weight biomass was required (Shaw et al., 2018), which was achieved by collecting approximately 50–100 living individuals per zinc case. Particulate organic matter (POM) smaller than 30 μm was determined in triplicate and per sample, as follows: a piece of mat was pressed gently against a Nytal sieve of 30-μm-mesh-size diameter. From this filtrate, and after standing for 1 h, the POM fraction was obtained with the heavier material that precipitated in 50-mL Corning tubes. Therefore, we obtained the fraction of organic matter that mostly contains exopolysaccharides (EPSs) from cyanobacterial community, but also organisms smaller than 30 μm. From the supernatant, and after filtering through a 0.22-μm hydrophilic membrane and concentrating by evaporation at 40°C under vacuum, dissolved organic matter (DOM) fraction was obtained and encapsulated. The isotopic ratios from all samples were analyzed by mass spectrometry (MEIR; 20-20 PDZ Europa mass spectrometer).

### DNA Extraction, Sequencing, and Ribotype Diversity Analysis

Total genomic DNA was extracted in triplicate for the studied microbial mats. The DNAeasy Power Biofilm kit (Qiagen) was used for DNA extraction, using the protocol supplied with the kit. Bacterial 16S rRNA marker gene was amplified from the cellular fractions using the set of primers 8F15B (5′-AGAGTTTGATCCTGG-3′) and 515R14AM (5-TTACCGCGGCTGCT-3′) (Aguirre de Cárcer et al., 2011). The pool of samples with the prepared libraries was sequenced by Illumina MiSeq platform.

Bacterial 16S rRNA gene diversity was assessed with QIIME v2-2019.4 (Bolyen et al., 2019). Briefly, cleaned and trimmed paired reads were filtered and denoised using DADA2 plug-in (Callahan et al., 2016). For chimera identification, 300,000 training sequences were used. Identified amplicon sequence variants (ASVs) were aligned using MAFFT (Katoh et al., 2002) and further processed to construct a phylogeny with fasttree2 (Price et al., 2010). Taxonomy was assigned to ASVs using the q2-feature-classifier (Bokulich et al., 2018) and blasted against the SILVA v132 99% 16S sequence database (Quast et al., 2012). Sequences assigned to chloroplasts were removed from the dataset.

Sequences generated by this study were deposited to GenBank under the BioProject accession number PRJNA795814.

### Data Analyses

Statistically significant differences between “penguin-impacted” and “macrofauna-free” microbial mats in terms of nutrient levels, diversity indices, and relative abundance of fungal biomass (ergosterol), primary producers (chla), and meiofaunal groups (tardigrades, nematodes, and rotifers) measurements were determined with a Welch *t*-test in R v4.1.1 (R Core Team, 2019). To further understand the relationships among environmental and chemical parameters with microbial mat communities, Pearson correlation coefficients among C, N, P, C/N, N/P, and δ^15^N content of the microbial mats, as well as latitude and the ‘number of days per year with temperatures above freezing’ (TAF) from LI, AI, CP, and BP sites, were calculated using PAST 4.03 software (Hammer et al., 2001). For inference, normal distributions were assumed, and two-tailed *t-*tests were calculated for the non-correlation null hypothesis, with 0.05 significance level.

To assess the trophic pathways within the microbial mat community, a Bayesian isotopic mixing model, available as an open-source R package, simmr (Parnell and Inger, 2016), was used. Mixing models use organism isotopes to estimate the proportional contribution of sources to the consumer diet composition (Parnell et al., 2010). The model was fitted via Markov Chain Monte Carlo method. After 200,000 iterations of the algorithm, we represented the marginal posterior dietary proportions with boxplots and considered the medians as single summary values. We assumed similar stoichiometry for C and N fractionation through the food web in the model. Trophic enrichment factors (TEFs) were based on mean trophic fractionations with standard deviations (TEF δ^13^C: −0.4 ± 1.3; TEF δ^15^N: −3.4 ± 1) (Post, 2002).

Alpha rarefaction analysis was performed using the “qiime diversity alpha-rarefaction” function, to determine if the samples were sequenced to a sufficient depth. Alpha diversity indices (richness and Shannon index) were estimated using the “qiime diversity core-metrics-phylogenetic” function. The lowest sample-specific sequencing depth (24,892) was used to compensate for the variation in read numbers. The Margalef diversity index (Margalef, 1958) was calculated by the formula: $d=\frac{\left(s-1\right)}{\text{ln}\,N}$, where *S* is the number of ASV, and *N* is the number of feature counts in the sample. Shannon and Margalef indices were measured to compare the diversity of bacteria among samples. Beta diversity was assessed using Bray–Curtis dissimilarities among the community compositions of the sampling sites and visualized with non-metric multidimensional scaling (NMDS) using the phyloseq package (v1.14.0) (McMurdie and Holmes, 2013) in R. A UPGMA cluster dendrogram based on Bray–Curtis dissimilarity was used to visualize similarities among the bacterial communities from the studied microbial mats. Differences among bacterial communities were tested with permutational multivariate analysis of variance (PERMANOVA) test using the vegan package (v2.5-3) (Oksanen et al., 2013) in R.

To explore the relationships between microbial communities and environmental variables, a two-distance–based redundancy analysis (db-RDA) was performed (Legendre and Anderson, 1999). The collinear or non-significant predictors were eliminated by using the “ordistep” analysis in the vegan package (Oksanen et al., 2013) in R. All significance testing was assessed by Monte Carlo permutation of full models with 999 unrestricted permutations. To examine the difference in relative abundance of microorganisms (family level) between penguin impacted and non-impacted locations, we calculated log_2_-fold changes using the DESeq2 package (v.1.34.0) (Love et al., 2014) in R.

## Results

### Nutrient Levels and Its Origin Differ Between “Penguin-Impacted” and “Macrofauna-Free” Microbial Mats

The lowest concentrations of P, and the highest C/N and N/P ratios were found in BP, the microbial mat far away from any penguin colony and/or macrofaunal activity (Table 1). The highest values of P, N, and the lowest C/N and N/P ratios were present in those microbial mats adjacent to an active penguin colony and considered as “penguin-impacted” samples (LI, AI, and CP). We observed significantly higher values when comparing “penguin-impacted” microbial mats with the sample without macrofaunal influence for P (*t* = 4.81, *p* < 0.01), C/N (*t* = −13.34, *p* < 0.01), and N/P ratios (*t* = −8.34, *p* = 0.01). BP microbial mat also displayed the lowest concentration of N-NH_4_^+^ and NO_3_, compared with “penguin-impacted” microbial mats (Table 1). Despite the high variation shown for δ^15^N signatures among LI, AI, and CP samples, statistical analysis showed that δ^15^N values at “penguin-impacted” samples were significantly higher than those in the “macrofauna-free” microbial mat (*t* = 8.46, *p* < 0.01).

**TABLE 1:** Mean (standard deviation in parentheses) values of the physicochemical characteristics of the four microbial mats included in this study.

	Lagotellerie Island (LI)	Avian Island (AI)	Cierva Point (CP)	Byers Plateau (BP)
	67°53′16″S 67°24′02″W	67°46′15″S 68°53′10″W	64°09′00″S 60°57′50″W	62°65′49″S 61°11′41″W
**Physicochemical characteristics**				
TAF (days per year)	83	83	13	190
C (mg⋅g^–1^)	115.6 (12.2)	285.9 (0.6)	190.3 (12.8)	212.9 (20.8)
N (mg⋅g^–1^)	21.3 (2.4)	44.9 (0.4)	26.3 (1.8)	21.1 (2.4)
P (mg⋅g^–1^)	33.7 (0.9)	57.2 (2.5)	11.0 (0.6)	1.8 (0.1)
δ^15^N (‰)	22.3 (0.2)	12.4 (0.1)	8.8 (0.1)	−3.9 (0.3)
C/N	5.4 (0.0)	6.4 (0.1)	7.2 (0.2)	10.1 (0.2)
N/P	0.6 (0.1)	0.8 (0.0)	2.4 (0.3)	12.0 (2.2)
NH_3_-NH_4_^+^ (mg⋅kg^–1^)	255	513	459	28
NO_3_ (mg⋅kg^–1^)	130	41	88	24
TOC (mg⋅cm^–2^)	3.9 (0.2)	4.9 (0.5)	3.4 (0.7)	5.8 (1.9)
Chla (μg⋅cm^–2^)	26.8 (0.3)	8.1 (1.0)	5.2 (1.1)	11.6 (1.7)
Fungal biomass (μg⋅cm^–2^)	109 (31.1)	61 (27.2)	37 (18.2)	9 (3.6)
**Meiofauna**				
Nematodes (ind⋅cm^–2^)/%	1.7 (3) / 0	22.9 (13) / 6	48.9 (34) / 52	96.7 (55) / 50
Tardigrades (ind⋅cm^–2^)/%	644.9 (588) / 88	169.3 (69) / 45	7.6 (11) / 8	70.3 (32) / 37
Rotifers (ind⋅cm^–2^)/%	106.9 (122) / 12	184.6 (29) / 49	37.7 (25) / 40	28.5 (15) / 13
**Bacterial diversity and richness**				
Richness	447 (73)	573 (49)	512 (58)	601 (47)
Shannon	7.3 (0.3)	7.8 (0.2)	7.2 (0.3)	7.8 (0.1)
Margalef	43.3 (6.5)	55.8 (4.3)	49.3 (5.3)	58.7 (4.1)

*Meiofaunal relative abundances estimations (per surface), richness (defined by the number of ASVs), Shannon and Margalef indices of the bacterial community were included.*

N and P concentrations in microbial mats were significantly positively correlated with penguin population in the surroundings (0.97 and 0.90, respectively) (Supplementary Figure 1). Therefore, local macrofauna, mainly represented by penguins, indirectly may contribute to a nutrient enrichment of the environment by providing nitrogen and phosphorus compounds to the studied microecosystems. C was also positively correlated with penguins (0.73), whereas TOC was correlated with TAF (0.68).

### Fungal Community Shows High Correlation With Penguin-Derived N and P

Fungal biomass, represented by ergosterol concentrations, was significantly higher in “penguin-impacted” microbial mats (*t* = 5.47, *p* < 0.01), showing an average 10-fold higher than in BP (Table 1). Moreover, the relative abundance of the fungal community showed a negative correlation with C/N and N/P ratios (−0.86 and −0.78, respectively) and a positive correlation with P concentration, δ^15^N values, and latitude (0.79, 0.86, and 0.91, respectively) (Supplementary Figure 1). No significant differences were observed for the relative abundance of photosynthetic primary producers, represented by chla concentration, among the samples (data not shown). Although a positive correlation with δ^15^N (0.61) and a negative correlation with C (−0.75) appeared. TOC, related to total biomass of cyanobacterial microbial mats, showed positive correlation with the climatic parameter of TAF (0.68).

The microscopic analyses indicated that the microbial community matrix was dominated by different Oscillatoriales and Synechococcales (Cyanobacteria) morphotypes (*sensu* Hoffmann et al., 2005). *Wilmottia* sp. (5.5–7 μm in diameter), previously classified as *Phormidium* (Anagnostidis and Komárek, 1988), was common in all the studied microbial mats. Morphotype I *sensu* Broady and Kibblewhite (1991) (2 μm in diameter, *Leptolyngbya cf. antarctica*), dominated the cyanobacterial community of BP, but also occurred in all the mats (Supplementary Figure 2). Abundant microcolonies (20–100 μm in diameter) of Nostocalean morphotypes (3 μm in diameter cells) appeared mostly in BP mat, below photosynthetically active layers. Unicellular cyanobacteria (e.g. *Synechococcus* spp.; 1.5 μm in diameter) were relatively abundant and occurred intermixed within the EPS matrix. The photosynthetic community also included Chlorophyta, although with an apparent relative lower abundance than the cyanobacterial community. *Prasiola* sp. constituted a top layer in AI and CP microbial mats, where *Chlamydomonadaceae* were also found. *Chlamydomonas* sp. (16–20 μm in diameter) was very common within the matrix of LI. Bacillariophyta was not abundant but observed in AI and LI samples. In all microbial mats, *Mougeotia* sp. (22–27 μm in diameter) occurred.

### The Meiofauna Groups Are Heterogeneously Distributed Among the Samples

The meiofauna found in the studied microbial mats consisted of tardigrades *Hypsibiidae*, bdelloid rotifers, and nematodes (Supplementary Figure 2). None of the groups in any of the samples was clearly associated with a specific layer or portion of the microbial mats. The relative abundance of tardigrades, rotifers, and nematodes (Table 1) was heterogeneous and not correlated with P- and N-related parameters, and no significant correlation was found among groups. Nonetheless, tardigrades were more abundant in LI and AI microbial mats (86 and 45% of total meiofaunal individuals captured per surface, respectively), counting dozens of individuals per analyzed sample (Table 1). Nematodes predominated in CP and BP microbial mats (52 and 50% of total meiofaunal individuals captured per surface, respectively). The relative abundance of bdelloid rotifers was 49 and 40% of total meiofaunal individuals captured per surface in AI and CP, remaining less than 15% in the other microbial mats.

### Trophic Relationships Change Among the Studied Microbial Mats

For all microbial mats, Cyanobacteria were the most abundant primary producers, and consequently a potential C source for the trophic web. Also, *Chlamydomonadaceae* (Chlorophyta) for CP and LI mats, *Prasiolaceae* (Chlorophyta) for AI mat, and mosses (BP mat) were included in the food web study. In CP and BP, δ^13^C of Cyanobacteria, POM, and DOM (Supplementary Table 1) showed signals similar to each other, whereas in the other mats, the sources showed different values of both stable isotopes. δ^13^C and δ^15^N signals in the BP microbial mat were clearly different from the isotopic signals found in the other mats. While ^15^N/^14^N values of BP were negative in all separated groups (with the exception of nematodes where it was slightly positive), in all the other microbial mats, δ^15^N was always clearly positive averaging from 8.8 to 22.3‰. The same pattern was observed in BP for ^13^C/^12^C values, showing less negative values in all the trophic groups studied compared with the other mats.

**FIGURE 2:**
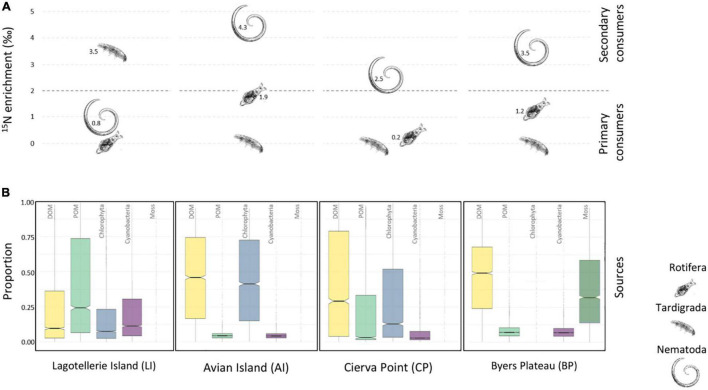
**(A)** Enrichment in δ^15^N of rotifers, nematodes, and *Hypsibiidae* tardigrade. For estimating the differences between “primary consumers” and “secondary consumers,” an enrichment of 2–4‰ was considered. **(B)** Simmr credibility interval plot of the contribution of potential food sources of the studied microbial mats (boxes enclose the 50% credibility interval; lines within boxes represent median values).

Consumer’s category consisted for all samples of tardigrades *Hypsibiidae*, rotifers, and nematodes. Tardigrades in LI microbial mat were classified as top consumers from the marked differences in the ^15^N signatures, showing δ^15^N values that exceed those of nematodes by 2.8 (Figure 2A). Nevertheless, nematodes in AI, CP, and BP mats were situated at the top of the food web, as concluded from the observed differences of 4.3, 2.5, and 3.5 δ^15^N with tardigrades, and differences of 2.5, 2.2, and 2.3 δ^15^N with rotifers. Enrichments in δ^15^N found between nematodes and rotifers for LI (0.8) and between rotifers and tardigrades for AI, CP, and BP (1.9, 0.2, and 1.2, respectively) remained below typical 2–4‰ difference among trophic levels.

To determine C pathways, we plotted the credibility intervals for the proportions of each potential source to the consumer’s diet contribution in each mat (Figure 2B). The Bayesian isotopic mixing model estimated which were the preferred C sources by consumers, showing DOM as the main food source in most of the microbial mats. Thus, the estimated dietary proportions for DOM in AI, CP, and BP were 46, 29, and 49%, respectively. For LI mat, the POM fraction appeared with the highest dietary proportion (24%) of available sources. Cyanobacterial fractions showed low proportions as a potential source in all microbial mats. Chlorophyta proportions from AI and CP (41 and 12%, respectively) placed them as the second potential food source, as mosses did in BP (29%).

### Bacterial Communities Differ Significantly in the Surroundings of Penguin’s Colonies

**FIGURE 3:**
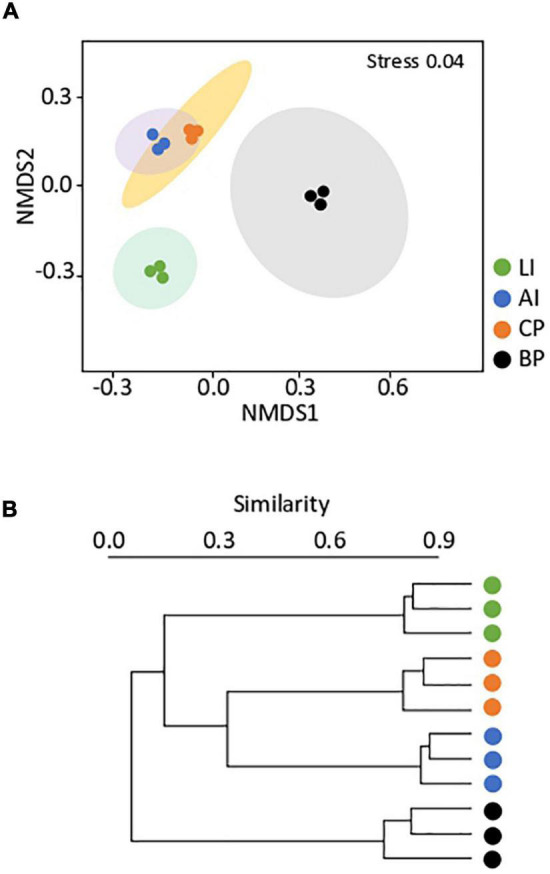
**(A)** Non-metric multidimensional scaling (NMDS) of the bacterial community composition of Lagotellerie Island (LI), Avian Island (AI), Caleta Primavera (CP), and Byers Peninsula Plateau (BP) microbial mats. The multivariate analysis is based on Bray–Curtis dissimilarity matrices between the microbiome profiles at an ASV level. Distances between symbols on the ordination plot reflect relative dissimilarities in community structures. Ellipses indicate 95% confidence intervals fitted into the spatial ordination. **(B)** UPGMA cluster dendrogram of the bacterial community within the microbial mats included in this study. The relative abundances were used to evaluate the relationships between bacterial communities, using weighted pair clustering based on Bray–Curtis measurements.

The total number of 16S rRNA gene sequences obtained was 1,393,079, which were grouped into 3,031 different ASVs. In order to analyze the similarities among bacterial communities inhabiting the microbial mats, the sequencing taxonomic abundance profiles (ASV level) were used to compute a Bray–Curtis dissimilarity matrix, summarized in two dimensions by using NMDS (Figure 3A). Samples were grouped according to location, showing greater dissimilarities among samples than among replicates within the same sample. Hierarchical clustering based on community structure similarity (Figure 3B) revealed four main clusters according to each sampling location. AI and CP mats clustered together, showing a percentage of similarity of 32%. BP showed the community the lowest percentage of similarity (∼7%) to the other microbial mats. A PERMANOVA test corroborated significant evidence of heterogeneity (*F* = 16.61, *p* < 0.01) for bacterial communities among microbial mats.

**FIGURE 4:**
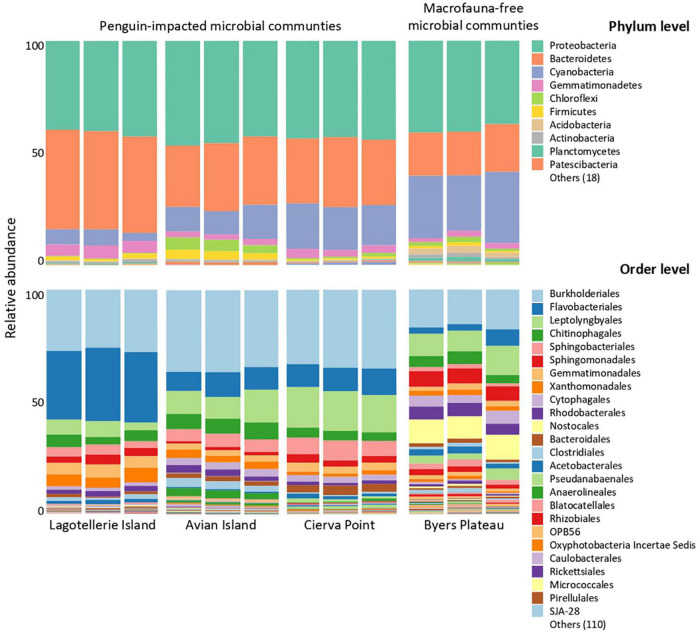
Relative abundance of 16S rRNA genes at different taxonomic resolution (phyla vs. order) from microbial mats included in this study.

Bacterial communities (Figure 4) were dominated by sequences assigned to Proteobacteria (37–47%) and Bacteroidetes (19–43%). Cyanobacteria sequences ranked as the second phylum in BP with the highest number of assigned sequences (27.8%) and third in the remaining samples (4–21%). When analyzing the sequences at a higher taxonomic resolution, important differences in the composition of the bacterial community appeared. The most abundant orders were Burkholderiales, Leptolyngbyales, and Flavobacteriales, followed by Sphingobacteriales, Chitinophagales, and Sphingomonadales. However, whereas these 6 taxa reached relative abundances of between 68 and 78% in the ‘penguin- affected’ microbial mats, in BP they did not reach 50%. Moreover, those taxa (order level) with relative abundances lower than 1% reached 11% of the total community sequences in BP, whereas in the other microbial mats they represented between 5 and 8% of the total.

The large variation (e.g. standard deviation) in values of the measured bacterial diversity indices among the samples indicated high spatial heterogeneity (Table 1). The lowest number of different ASVs was found in LI mat (367), as well as the lowest values for Shannon and Margalef indices (7.0 and 36.0, respectively). The highest number of different ASVs (655), as well as the highest values for Shannon and Margalef indices (7.9 and 63.4), was shown for BP microbial communities. We observed significantly higher values when comparing the microbial mat without animal influence with microbial mats associated with “penguin-impacted” environments for richness (*t* = −2.42, *p* = 0.05), Shannon (*t* = −2.61, *p* = 0.03), and Margalef indices (*t* = −2.67, *p* = 0.04). In addition, bacterial richness and Margalef index values were positively correlated with C (0.64 and 0.66, respectively) and C/N (0.60 and 0.62, respectively) and negatively correlated with δ^15^N (−0.67 and −0.69, respectively). Shannon index appeared positively correlated with TAF (0.63).

We identified 29 bacterial families that changed significantly with the presence of penguins using DeSeq2, with 25 that decline and 4 that increase (Supplementary Table 2). Five of the 25 taxa were related to cyanobacterial families (Cyanobacteriaceae, Pseudanabaenaceae, Coleofasciculaceae, Leptolyngbyaceae, and Nostocales incertae sedis), whereas Bacteroidetes and Proteobacteria were the predominant phyla, showing the largest number of families involved (7 and 6, respectively). Flavobacteriaceae, Crocinitomicaceae, and Rhodothermaceae were more abundant in “penguin-affected” microbial mats. The most important factors within the physicochemical environment that directly or indirectly affected the bacterial community composition of microbial mats, based on the distance-based redundancy analysis (db-RDA), were NO_3_^–^ (*F* = 10.61, *p* = 0.01), N-NH_4_^+^ (*F* = 14.76, *p* = 0.01), and δ^15^N (*F* = 12.94, *p* = 0.00). These factors together contributed significantly to the variance (∼85%, *p* < 0.01).

## Discussion

### Effects of Penguin Droppings on Nutrient Levels of the Microbial Mats

In the absence of biological activity, the Antarctic terrestrial ecosystems exhibit an oligotrophic or ultraoligotrophic status (Tanabe et al., 2017), as the ability of non-biological processes to transport nutrients is very limited (Wang et al., 2020). Under these circumstances, any nutrient input into the system will be of significance for the community as most of the ecosystems are usually N (Davey, 1993a,b; Hawes et al., 1993) and P limited (Laybourn-Parry and Wadham, 2014). Seabirds are the main exchangers of nutrients between marine and terrestrial systems (De La Peña-Lastra, 2021), and their presence could deeply modify the ecosystems around their colonies (Otero et al., 2018). According to our results, microbial mats thriving in the vicinity of a penguin colony showed the highest concentrations of P and inorganic N (N-NH_4_^+^ and NO_3_^–^), which constitute the main nutrients in fresh seabird feces after the decomposition of uric acid (Schmidt et al., 2004). Moreover, a positive correlation was shown between the concentration of TN and P and the presence of penguins. These data suggest that penguins and other seabird droppings represent an input of nutrients for the studied microbial mats, as previously reported for different terrestrial polar ecosystems (Jakubas et al., 2008; Zmudczyñska et al., 2012; Ball et al., 2015; Zawierucha et al., 2016, 2019; Bokhorst et al., 2019; Wang et al., 2020; Ramírez-Fernández et al., 2021) and probably constitute the most important source of nutrients in these microecosystems.

Nutrient levels within microbial mats are only partially related to the surrounding waters in streams and/or ponds, given the rapid dynamics of elements within the microbial mats (e.g. interstitial water steep gradients) (Wood et al., 2015). The ecological stoichiometry referred to in ratios (e.g. C/N or N/P) focuses on the relationships between organism, ecosystem structure, and function with the environment (Elser et al., 2000). According to our results, C/N and N/P ratios were significantly lower in LI, AI, and CP samples compared with BP mat, grown in a nutrient-poor area, far from the coast and geographically isolated from marine vertebrates. Therefore, microbial mats from penguin-impacted locations were related to a more eutrophic status (based on the Redfield Ratio C:N:P), compared with a more common oligotrophic status displayed by BP. These differences in the trophic status could be associated with processes of “ornitheutrophication,” previously described for soils and waters where environmental conditions were altered because of the establishment of breeding colonies of seabirds (Otero et al., 2018). Moreover, elemental ratios can be related to important ecological features such as ecosystem-specific composition and diversity (Venterink et al., 2003), processes of organic matter decomposition (Güsewell and Gessner, 2009), and with the ability of organisms to adapt to environmental stresses (Woods et al., 2003). Therefore, the differences observed in nutrient levels would have consequences in the entire biological community that constitute these microecosystems.

Our results also evidenced that ^15^N values were significantly higher in penguin-impacted microbial mats. These data are consistent with previous studies in other Antarctic and sub-Antarctic regions, where δ^15^N values of cryptogams (Erskine et al., 1998; Bokhorst and Convey, 2016) and microbial mats (Wang et al., 2020) decreased with increasing distance from penguin colonies. Crittenden et al. (2015) showed that NH_3_ emitted from a penguin colony at Cape Hallett had positive δ^15^N values, reflecting ^15^N enriched in the penguin guano and impacted soils. This may be related to increases in marine N source due to a higher trophic position of seabirds (Cherel et al., 2007) and preferential volatilization of ^14^N from guano (Mizutani et al., 1985). But higher values of ^15^N were found throughout the entire food web of LI, AI, and CP mats, as previously reported when studying the impact of penguin guano on terrestrial and aquatic Antarctic ecosystems (Bokhorst and Convey, 2016; Bokhorst et al., 2019; Wang et al., 2020). Therefore, N derived from penguins and other marine vertebrates moves through food webs from primary producers to consumers, and all the organisms would be directly linked to the marine-derived δ^15^N signature.

### Impact of Penguin-Derived Nutrients on the Food Webs and Trophic Relationships of Microbial Mats

The consequences of nutrient load into the terrestrial ecosystem, mainly by penguins but also by other seabirds and marine mammals, may become more evident in primary producers than in consumers, as typically N and P limit primary production in marine and terrestrial environments (Chadwick et al., 1999; Mills et al., 2004). However, synergistic interactions between limited supplies of these nutrients are widespread across aquatic and terrestrial systems (Elser et al., 2007), and nutrient colimitation and other kinds of interactions between limiting resources also need to be considered (Gleeson and Tilman, 1992; Danger et al., 2008; Harpole et al., 2011). Allochthonous nutrient enrichment of Antarctic lakes by local fauna has been documented, reporting increases in chlorophyll concentrations due to these processes (Hawes, 1990; Vinocur and Unrein, 2000; Izaguirre et al., 2001; Rochera, 2012). In the studied cyanobacterial microbial mats, chla concentrations varied in the samples within the range of the values reported in previous studies from Antarctica (Hawes et al., 2019), with more than a two-fold increase between LI and BP. Also, a positive correlation appeared between δ^15^N and chla concentration. Therefore, nutrient levels may influence the activity and growth of primary producers in these forced ecosystems. However, the length of the season with liquid water available, referred to in this study as the number of days per year with temperatures above the freezing point (TAF), could be relevant by limiting the growth, as suggested by Billi and Potts (2002) even in nutrient-replete ecosystems, thus explaining the positive correlation shown between TOC and TAF.

Understanding the structure of the food web is necessary to determine how organisms and the environment interact and therefore the functioning of the ecosystem (Perkins et al., 2014), but there are few articles published on these interactions in microbial mats. According to our results, the trophic structure of the microbial mats was different among locations, with consumers appearing in different abundances and trophic positions within the studied food webs. Our data indicated that tardigrades and rotifers appeared by dozens in samples taken in the vicinity of penguin colonies and related to more eutrophic conditions, whereas nematodes proportionally dominated BP, considered in more oligotrophic status. These differences shown in their distribution could be partially revealed by the biotic interactions among the meiofaunal groups, as suggested for rotifers and *Panagrolaimus* sp. on penguin-impacted soils (Porazinska et al., 2002; Sohlenius et al., 2004). However, our results did not show a significant correlation to support the co-occurrence among the studied taxa, although the taxonomic diversity within each group was not considered. It has been also suggested that primary producer populations affect the abundance and diversity of higher trophic levels (Bokhorst et al., 2015; Zawierucha et al., 2019), which in turn represent an important driver of their populations by grazing and nutrient recycling (Zawierucha et al., 2018). The most widespread tardigrade genera in Antarctica (*Acutuncus, Hypsibius*, and *Isohypsibius*) are defined as grazers (Nelson et al., 2018). Thus, higher densities of grazers at penguin-impacted microbial mats may be explained by the availability of other resources such as green algae (Vonnahme et al., 2016), as suggested by increases in chla concentration, and probably higher abundances of fungi (Zwolicki et al., 2016; Zawierucha et al., 2019), as suggested by increases in ergosterol concentration. Fungi, with a key role as decomposers within the mat community (Velázquez et al., 2017), also appeared highly positively correlated with P and δ^15^N and highly negatively correlated with C/N and N/P. These data agree with previous studies, where seabirds-derived nutrients condition the growth rate and diversity of fungal populations (Osono et al., 2006). Therefore, our results suggest that penguin-derived nutrients condition trophic community within microbial mats at two levels: through derived remains (carcasses, pellets, feathers, eggs, etc.) that provide substrates for decomposers and detritivores, and through facilitating the development of populations of herbivores through the fertilizing effects of excrements on primary producers.

Based on the potential food sources provided by the model and the feeding ecological strategies of consumers, our results suggest that the differences in δ^15^N may be related to an enrichment of δ^15^N for rotifers intakes caused by preferential consumption of DOM and/or bacteria, and for tardigrades caused by ingesting POM and algae, as previously suggested in microbial mats from Antarctica (Velázquez et al., 2017; Almela et al., 2019). Data on feeding preferences of Antarctic rotifers (bdelloids), which are filter feeders, suggested that the diet consisted of dead organic matter and unicellular algae (Iakovenko et al., 2015), although they could probably feed on bacteria (Jaroměřská et al., 2021). In the studied microbial mats, POM fraction would be composed of EPS, detritus, bacteria, and ciliates, but we also found abundant unicellular cyanobacteria (approximately 1.5 μm in diameter) forming this matrix. As nitrogen-fixing organisms, such as cyanobacteria, have high content of proteins and high ^15^N values (Kohler et al., 2018), tardigrades from LI mat feeding on them would consequently present higher ^15^N compared with rotifers, as it has been suggested for tardigrades in Arctic cryoconite holes (Jaroměřská et al., 2021). Fungi should also be considered as a potential food source for these herbivores (Darby and Neher, 2016), although their role within the trophic relationships could not be studied. In BP mat, tardigrades probably feed on POM, which would contain moss fragments, present in the surroundings but rarely within microbial mats. Because cyanobacteria are such a significant portion of the microbial mats, we expected a trophic relationship with nematodes, as shown in different ecosystems (Darby and Neher, 2016; Andriuzzi et al., 2018). However, nematodes did not appear trophically related to cyanobacteria and probably feed on bacteria (Nielsen et al., 2011; Caruso et al., 2019) and other consumers, as suggested by Almela et al. (2019). Therefore, the shift in POM composition could explain the isotopic differences between tardigrades and rotifers, which in turn would be related to the availability of nutrients and the presence of different primary producer densities in the studied microbial mats. These results also suggest that much of the C processed by invertebrate food webs is derived from detrital resources, as described by Koltz et al. (2018) for the Alaskan tundra ecosystem. Furthermore, the differences shown in δ^15^N among consumers suggest that tardigrades positioned at the top of the trophic web in LI mat, with a typical enrichment of 2–4‰ from diet to consumer (Post, 2002; Vanderklift and Ponsard, 2003; Caut et al., 2009). In the remaining samples, which includes “penguin-impacted” and “macrofauna-free” mats, nematodes were the top consumers, as shown for Antarctic soils (Shaw et al., 2018). The differences in ^15^N observed in tardigrades could also suggest a different trophic level in LI for *Hypsibiidae* specimens, or a higher ^15^N enrichment of this group by feeding directly on marine-derived δ^15^N enriched primary producers in comparison with the other consumers, which could act mainly as detritivores. We suggest that further research is needed to better understand species diversification and co-occurrence through the different levels of consumers to evaluate in detail the alterations of meiofaunal groups due to penguin-derived nutrients in microbial mats.

### Consequences of Penguin-Derived Nutrients on Microbial Community Composition and Bacterial Diversity

Our analysis revealed that inorganic nitrogen (N-NH_4_^+^ and NO_3_^–^) is a key factor in driving bacterial community composition, which agree with previous studies of polar microbial communities from terrestrial ecosystem (Kleinteich et al., 2017; Valdespino-Castillo et al., 2018; Dillon et al., 2020; Jackson et al., 2021). A similar pattern was determined for Antarctic soils (Smith et al., 2010), where NH_3_, NO_3_^–^, and total N, among other physicochemical parameters, were driving the microbial community composition. While P has been shown as the most limiting nutrient in processes for microbial autotrophs in glacial retreat areas (Darcy et al., 2018), it is well known that N is a key nutrient in ecosystems, and its availability is correlated with the presence of marine vertebrates (Bokhorst et al., 2019; Wang et al., 2020). Our results showed that penguin-derived nutrients influence the community composition of microbial mats with significant differences between “penguin-impacted” and “macrofauna-free” samples. Bacteria belonging to the order Clostridiales and Flavobacteriales had higher relative abundances in those microbial mats influenced by penguins, as was reported in ornithogenic soils (Santamans et al., 2017; Guo et al., 2018) and lakes frequented by different marine vertebrates in Antarctica (Picazo et al., 2019). At family level, Flavobacteriaceae, Crocinitomicaceae, Rhodothermaceae, and Xanthomonadaceae were significantly more abundant in samples affected by penguins. Some of these taxa have been associated with soils influenced by the presence of different macrofaunal groups (Ramírez-Fernández et al., 2021) and are also recognized as initial metabolizers of labile C inputs in soils (Padmanabhan et al., 2003) during initial stages of the microbial loop. Different genera from these families, which were also found in our samples (e.g. *Flavobacterium* and *Gelidibacter*), are common members of penguin gastrointestinal tract microbiota (Barbosa et al., 2016). Therefore, these results suggest that macrofaunal groups directly influence the bacterial composition of Antarctic microbial mats. This could determine the internal nutrient cycling processes within microbial mats and potentially explain the relationships between their bacterial communities and the nutrient content.

Cyanobacteria ranked as the second phylum with the highest number of assigned sequences only in BP mat, showing lower relative abundances in penguin-impacted samples. These differences were observed when comparing oligotrophic to nutrient-rich lakes in Antarctica, suggesting that high N concentration associated with enrichment by marine vertebrates would tend to favor non-cyanobacterial taxa (Pearce et al., 2005). Significant differences among penguin-impacted and non-impacted microbial communities were also found for Cyanobacteria at family level. According to our results, sequences affiliated to the nitrogen-related taxa, such as Pseudanabaenaceae and Nostocales, were more abundant in macrofauna-free mat. Pseudanabaena and Geitlerinema potentially fix N_2_ into NH_4_^+^-NH_3_ (Bergman et al., 1997; Fernández-Valiente et al., 2007; Grim and Dick, 2016), and nitrifying bacteria, such as *Nitrospira* found in BP, oxidize it into NO_2_^–^ and NO_3_^–^, increasing the availability of nitrogen to the community. This would be the main N input pathway in mats from oligotrophic environments, as assimilating ^15^N-depleted abiogenic N results in negative δ^15^N values (Wang et al., 2020), as shown in BP. Marine vertebrates breeding areas, especially penguin rookeries, are considered nutrient hotspots, as a large proportion of the excreted N and P is present in highly bioavailable forms (Otero et al., 2018). The uric acid contained in the feces of these seabirds serves as a substrate for its mineralization in different forms of N through ammonification and nitrification processes. Nostocales activity in non-enrichment environments may constitute up to 23% of the assimilated nitrogen by mats community (Fernández-Valiente et al., 2007). But considering that N would be widely available in areas with local macrofauna, especially when penguins occur (De La Peña-Lastra, 2021), diazotrophs, such as Nostocales, may be less competitive in these communities, and consequently, their relative abundance would decrease. These results suggest that the presence of penguins and other seabirds, through the nutrients derived from its biological activity, has a significant influence on bacterial community composition and a strong influence on the nutrient cycle, affecting N utilization strategies of microbial mats.

Our study showed that richness and diversity of bacterial communities from Antarctic microbial mats are significantly influenced by the presence of nutrients derived from penguin activity. Thus, a lower number of bacterial species appeared in those mats from “penguin-impacted” locations and related to environments associated with more eutrophic conditions. These results are consistent with Pearce et al. (2005), where nutrient-enriched lake, due to seal activity, showed reduced species richness in comparison to more oligotrophic lakes. This reduction in bacterial species richness was also accompanied by an increase in evenness among key groups. In proportion to the overall population size, Burkholderiales, Flavobacteriales, and Leptolyngbyales sequences explained 31% of the bacterial community in BP, whereas in LI and CP, mats explained 55–65% of total sequences. Also, while in “penguin-impacted” mats, the taxa (order level) with relative abundances lower than 1% represented between 5 and 8% of the total, in BP it reached 11%. Nutrient recycling by bacteria might be extremely relevant for microbial mat survival and regeneration, in an ecosystem where the input of allochthonous organic C is limited due the predominant unproductive surrounding catchment (Lyons et al., 2013). A greater number of families mainly associated with Proteobacteria and Bacteroidetes, but also with Firmicutes and Acidobacteria, appeared significantly associated with BP. These groups have been classified as heterotrophic microbes and are major players in microbial communities around the Globe (Rasuk et al., 2016; Echenique-Subiabre et al., 2018; Valdespino-Castillo et al., 2018), with a key role in organic matter decomposition processes in Arctic soils (Tao et al., 2020). Also, the presence of Cyanobacteria and Chloroflexi families significantly associated with these nutrient-poor ecosystems, which can utilize different portions of the radiation spectrum for photosynthesis (Ley et al., 2006), suggests the importance of the autotrophic within this communities. Therefore, there is an apparent change in dominance related to penguin-derived nutrients, which, together with species richness, induces oligotrophic systems into communities previously associated with low resistance to environmental change, but related to a higher metabolic versatility.

## Conclusion

We conducted a comprehensive study on the impact of penguins on microbial mat communities. Using the penguins as an example on polar microbial mat communities, this study illustrates how nutrients derived from marine vertebrates can change community composition and trophic structure of the microbial mats in Antarctica. Our results showed that P concentrations, C/N and N/P ratios, and δ^15^N values of “penguin-impacted” microbial mats were significantly higher than values obtained for “macrofauna-free” sample, indicating a strong influence of their presence in these communities. Nutrients derived from penguin colonies and other marine vertebrates influenced the diversity and structure of bacterial communities, even affecting N utilization strategies of microbial mats. Furthermore, trophic interactions of communities within microbial mats resulted altered, as well as the relative abundance and trophic position of meiofaunal groups. This study contributes to the understanding of the biocomplexity of microbial mats, one of the most diverse ecosystems in non-marine Antarctica, by focusing not only on microbial communities but also on meiofauna. Our results are also relevant in the panorama of global change as nutrient availability in the ecosystem could change and modify the ecological relationships within the ecosystem, throughout alterations in the local fauna. Studies considering the taxonomy of meiofaunal groups would help to better understand the trophic relationships that take place in Antarctic cyanobacterial-based microbial mats.

## Data Availability Statement

The datasets presented in this study can be found in online repositories. The names of the repository/repositories and accession number(s) can be found below: https://www.ncbi.nlm.nih.gov/genbank/, PRJNA678484.

## Author Contributions

PA, AJ, and AQ collected the samples. PA analyzed the experimental data and wrote the initial manuscript. All authors designed the study and contributed to elaborate the final manuscript.

## Conflict of Interest

The authors declare that the research was conducted in the absence of any commercial or financial relationships that could be construed as a potential conflict of interest.

## Publisher’s Note

All claims expressed in this article are solely those of the authors and do not necessarily represent those of their affiliated organizations, or those of the publisher, the editors and the reviewers. Any product that may be evaluated in this article, or claim that may be made by its manufacturer, is not guaranteed or endorsed by the publisher.

## Supplementary Material

Supplementary Figure 1 Correlogram for the independent variables identified as relevant to explain the differences in the microbial mats. Colors of circles indicate the strength and direction of correlation. Statistical significance was inferred for *p* < 0.05.

Supplementary Figure 2 Light microscopy and fluorescence micrographs of the main primary produces and consumers within the food webs. **(A)** Chlamydomonadaceae (green algae) visualized in LI. **(B)** Nostocalean morphotypes (cyanobacteria) from BP mat. Oscillatoria filaments (cyanobacteria) visualized in BP with light microscopy **(C)** and fluorescence microscopy **(D)**. Black and white triangles indicate the presence of *Leptolyngbya* sp. filaments (cyanobacteria). **(E)** *Leptolyngbya* sp. filaments (cyanobacteria) in BP. **(F)** *Prasiola* sp. (green algae) visualized in AI. **(G)** *Hypsibiidae* tardigrades, **(H)** rotifers, and **(I)** nematode found in CP.

Supplementary Table 1 Description of samples and isotopic values (δ^13^C and δ^15^N) of sources and consumers included in trophic web analysis. Isotopic values are presented as per mille (‰) and related to the international standards of the Pee Dee Belemnite for carbon and atmospheric N_2_ for nitrogen. Standard deviation (SD) is shown between parentheses.

Supplementary Table 2 DESeq analysis of ASVs (family level) comparing the different microbial mats. Families significantly different in abundance between “penguin-affected” and “macrofauna-free” microbial mats are shown.
