# Supplementary figures and images for: Marine Vertebrates Impact the Bacterial Community Composition and Food Webs of Antarctic Microbial Mats
Source: Front Microbiol. 2022 Apr 8;13:841175. doi: 10.3389/fmicb.2022.841175 (PMC9023888; doi:10.3389/fmicb.2022.841175)

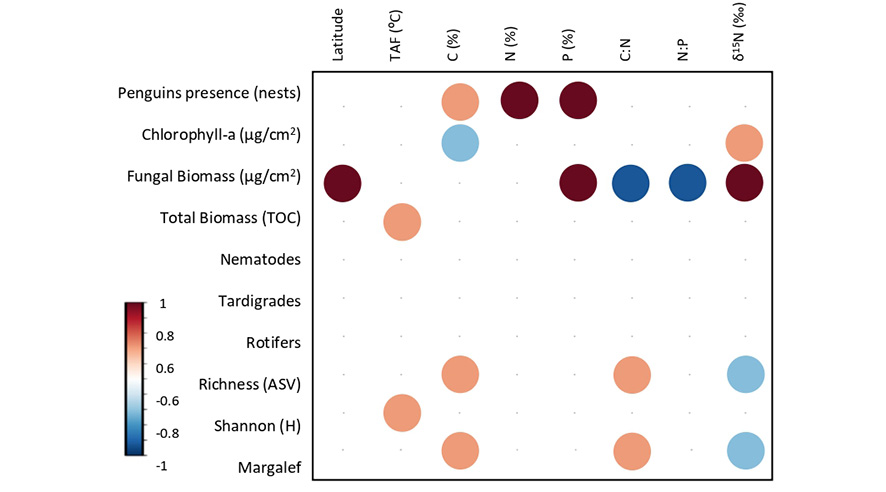

Supplement: Supplementary Figure 1 — Correlogram for the independent variables identified as relevant to explain the differences in the microbial mats. Colors of circles indicate the strength and direction of correlation. Statistical significance was inferred for p < 0.05. [file Image_1.JPEG]

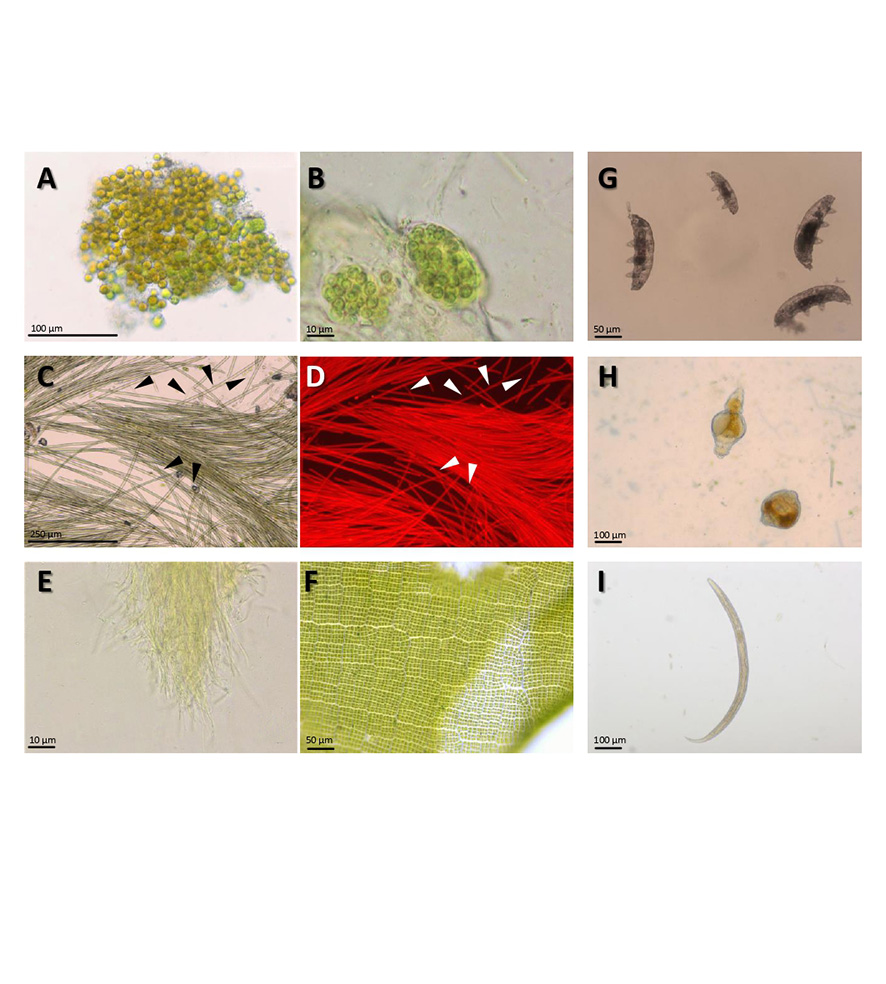

Supplement: Supplementary Figure 2 — Light microscopy and fluorescence micrographs of the main primary produces and consumers within the food webs. (A) Chlamydomonadaceae (green algae) visualized in LI. (B) Nostocalean morphotypes (cyanobacteria) from BP mat. Oscillatoria filaments (cyanobacteria) visualized in BP with light microscopy (C) and fluorescence microscopy (D). Black and white triangles indicate the presence of Leptolyngbya sp. filaments (cyanobacteria). (E) Leptolyngbya sp. filaments (cyanobacteria) in BP. (F) Prasiola sp. (green algae) visualized in AI. (G) Hypsibiidae tardigrades, (H) rotifers, and (I) nematode found in CP. [file Image_2.JPEG]
