# Supplementary material for: Marine Vertebrates Impact the Bacterial Community Composition and Food Webs of Antarctic Microbial Mats
Source: Front Microbiol. 2022 Apr 8;13:841175. doi: 10.3389/fmicb.2022.841175 (PMC9023888; doi:10.3389/fmicb.2022.841175)
Supplement: Supplementary Table 1 — Description of samples and isotopic values (δ13C and δ15N) of sources and consumers included in trophic web analysis. Isotopic values are presented as per mille (‰) and related to the international standards of the Pee Dee Belemnite for carbon and atmospheric N2 for nitrogen. Standard deviation (SD) is shown between parentheses. [file Table_1.docx]

|  | **Lagotellerie island** | | **Avian Island** | | **Cierva Point** | | **Byers Plateau** | |
| --- | --- | --- | --- | --- | --- | --- | --- | --- |
|  | δ^13^C (SD) | δ^15^N (SD) | δ^13^C (SD) | δ^15^N (SD) | δ^13^C (SD) | δ^15^N (SD) | δ^13^C (SD) | δ^15^N (SD) |
| **Sources** | | | | | | | | |
| Cyanobacteria  (filamentous) | -21.94 (0.8) | 25.09 (2.4) | -23.06 (0.2) | 10.42 (0.43) | -26.73 (0.1) | 9.22 (0.16) | -13.91 (0.5) | -4.91 (0.6) |
| [Chlorophyta](https://en.wikipedia.org/wiki/Chlorophyta) | -24.00 (0.5) | 24.81 (0.5) | -25.74 (0.5) | 10.9  (0.3) | -28.96 (0.6) | 6.45  (1.2) | _ | _ |
| Moss | _ | _ | _ | _ | _ | _ | -20.54 (0.0) | -2.67 (0.7) |
| POM | -18.21 (0.1) | 22.75 (0.1) | -26.64 (0.1) | 12.23 (0.1) | -26.19 (0.0) | 8.64  (0.2) | -14.39 (0.1) | -3.37 (0.1) |
| DOM | -20.63 (0.1) | 24.36 (0.2) | -26.01 (0.4) | 12.25 (0.1) | -25.05 (0.5) | 8.94  (0.1) | -13.70 (0.1) | -2.56 (0.2) |
| **Consumers** | | | | | | | | |
| Tardigrades (*Hypsibiidae*) | -23.58 (0.1) | 28.34 (1.9) | -27.77 (0.0) | 12.21 (0.5) | -27.36 (0.2) | 11.46 (0.3) | -15.26 (0.4) | -1.31 (0.4) |
| Rotifers | -23.10 (0.8) | 24.83 (1.1) | -28.46 (0.4) | 14.08 (0.6) | -26.29 (0.2) | 11.70 (0.1) | -17.18 (0.5) | -0.08 (0.4) |
| Nematodes | -24.09 (0.5) | 25.59 (1.4) | -25.31 (0.2) | 16.53 (0.8) | -26.52 (0.4) | 13.96 (0.9) | -13.94 (0.3) | 2.23  (0.7) |

**Table S1.**
