# Supplementary material for: Marine Vertebrates Impact the Bacterial Community Composition and Food Webs of Antarctic Microbial Mats
Source: Front Microbiol. 2022 Apr 8;13:841175. doi: 10.3389/fmicb.2022.841175 (PMC9023888; doi:10.3389/fmicb.2022.841175)
Supplement: Supplementary Table 2 — DESeq analysis of ASVs (family level) comparing the different microbial mats. Families significantly different in abundance between “penguin-affected” and “macrofauna-free” microbial mats are shown. [file Table_2.docx]

|  |  | **Phylum** | **Family** | **log2FoldChange** | **padj** |
| --- | --- | --- | --- | --- | --- |
|  | **‘Macrofauna-free’ microbial mat** | Acidobacteria | Holophagaceae | 20.683 | 0.000 |
|  |  | Bacteroidetes | PHOS-HE36 | 20.712 | 0.000 |
|  |  |  | Bacteroidetes_vadinHA17 | 9.480 | 0.001 |
|  |  |  | 37-13 | 6.852 | 0.000 |
|  |  |  | Microscillaceae | 4.269 | 0.004 |
|  |  |  | Prolixibacteraceae | 3.645 | 0.000 |
|  |  |  | Lentimicrobiaceae | 2.581 | 0.023 |
|  |  |  | Spirosomaceae | 1.580 | 0.001 |
|  |  | Chloroflexi | Chloroflexaceae | 8.031 | 0.004 |
|  |  |  | Anaerolineaceae | 7.353 | 0.002 |
|  |  |  | Roseiflexaceae | 4.961 | 0.044 |
|  |  | Cyanobacteria | Cyanobacteriaceae | 25.395 | 0.000 |
|  |  |  | Nostocales Incertae Sedis | 22.156 | 0.000 |
|  |  |  | Pseudanabaenaceae | 11.379 | 0.000 |
|  |  |  | Coleofasciculaceae | 8.508 | 0.011 |
|  |  |  | Leptolyngbyaceae | 1.256 | 0.028 |
|  |  | Fibrobacteres | Fibrobacteraceae | 21.238 | 0.000 |
|  |  | Firmicutes | Ruminococcaceae | 6.551 | 0.013 |
|  |  | Planctomycetes | Rubinisphaeraceae | 7.861 | 0.000 |
|  |  |  | Gemmataceae | 7.180 | 0.023 |
|  |  | Proteobacteria | Chitinibacteraceae | 8.096 | 0.003 |
|  |  |  | Beijerinckiaceae | 7.623 | 0.003 |
|  |  |  | A0839 | 5.344 | 0.026 |
|  |  |  | Rhodocyclaceae | 5.092 | 0.000 |
|  |  |  | Oligoflexaceae | 3.600 | 0.002 |
|  |  |  | Rhodanobacteraceae | 1.774 | 0.002 |
|  | **‘Penguin-affected’ microbial mats** | Bacteroidetes | Flavobacteriaceae | -1.797 | 0.011 |
|  |  |  | Crocinitomicaceae | -3.614 | 0.000 |
|  |  |  | Rhodothermaceae | -5.792 | 0.005 |
|  |  | Proteobateria | Xanthomonadaceae | -2.176 | 0.003 |
